# Antihyperglycemic, Antiaging, and *L. brevis* Growth-Promoting Activities of an Exopolysaccharide from *Agrobacterium* sp. FN01 (Galacan) Evaluated in a Zebrafish (*Danio rerio*) Model

**DOI:** 10.3390/foods13172729

**Published:** 2024-08-28

**Authors:** Xiaoqing Xu, Lingling Du, Meng Wang, Ran Zhang, Junjie Shan, Yu Qiao, Qing Peng, Bo Shi

**Affiliations:** 1 Feed Research Institute, Chinese Academy of Agricultural Sciences, No. 12 South Zhongguancun Street, Beijing 100081, China; xuxiaoqing@caas.cn (X.X.); qiaoyu@caas.cn (Y.Q.); pengqing@caas.cn (Q.P.); 2 Chengdu Sydix Biotech Co., Ltd., Building 1A, Chengdu Hi-Tech Incubation Park, No. 1480 Tianfu Avenue North, Hi-Tech Zone, Chengdu 610095, China; lynn@sydix.com.cn (L.D.); wangmeng@sydix.com.cn (M.W.); charles@sydix.com.cn (R.Z.); 3 Academy of Military Medical Sciences Institute of Pharmacology and Toxicology, Beijing 100039, China; shanjunjie001@126.com

**Keywords:** exopolysaccharide, *Agrobacterium* sp. FN01, antihyperglycemic, antiaging, *L. brevis* growth

## Abstract

*Agrobacterium* sp. are notable for their ability to produce substantial amounts of exopolysaccharides. Our study identified an exopolysaccharide (Galacan, 4982.327 kDa) from *Agrobacterium* sp. FN01. Galacan is a heteropolysaccharide primarily composed of glucose and galactose at a molar ratio of 25:1. The FT-IR results suggested that Galacan had typical absorption peaks of polysaccharide. The results of periodate oxidation, Smith degradation, and NMR confirmed the presence of structural units, such as β-D-Galp(→, →3)β-D-Galp(1→, →2,3)β-D-Glcp(1→, β-D-Glcp(1→, and →2)β-D-Glcp(1→. Galacan demonstrated significant biological activities. In experiments conducted with zebrafish, it facilitated the proliferation of *Lactobacillus brevis* in the intestinal tract, suggesting potential prebiotic properties. Moreover, in vivo studies revealed its antihyperglycemic effects, as evidenced by significant reductions in blood glucose levels and enhanced fluorescence intensity of pancreatic β cells in a streptozotocin (STZ)-induced hyperglycemic zebrafish model. Additionally, antiaging assays demonstrated Galacan’s ability to inhibit β-galactosidase activity and enhance telomerase activity in a hydrogen peroxide (HP)-induced aging zebrafish model. These findings emphasized the potential of Galacan as a natural prebiotic with promising applications in diabetes prevention and antiaging interventions.

## 1. Introduction

Considering the roles of polysaccharides in various life processes and their therapeutic potential and relatively low toxicity, polysaccharides are highly important in the healthcare, food, and cosmetic industries [1]. The microbial exopolysaccharides (EPS) that have been reported or are insufficiently researched are mostly due to their functional activities, which are useful for the development of food and pharmaceutical industries [2]. Among these, curdlan is a bioactive microbial exopolysaccharide that is composed of D-glucose with β-(1,3)-glycosidic linkages and is typically produced by *Agrobacterium* sp., *Rhizobium* sp., *A. faecalis*, *Gluconacetobacter xylinus*, *Bacillus cereus*, and other microorganisms [3,4,5]. *Agrobacterium* spp., such as *Agrobacterium* sp. ATCC31749 and *Agrobacterium* sp. CGMCC 11546, are typical bacteria that can produce a high amount of curdlan [6,7]. The name “curdlan” is attributed to its unique characteristic of “coagulating” when heat-treated, such as the heat-resistant gel formation, which is stable at 80~100 °C [1]. Oligosaccharides or derivatives obtained from curdlan are the most studied and exhibit immunomodulatory activities in humans and animals, as well as antibacterial, antitumor, anti-HIV, anti-dengue virus, and antioxidant properties [4,8].

Intestinal health is helpful to maintain the health of the host, and the attention is gradually increasing. EPS has been reported as able to be used as a novel prebiotic to regulate the intestinal microbiota and may help prevent and treat intestinal diseases [9]. EPS from *Lactobacillus pentosus* YY-112 can reduce the relative abundance of *Escherichia–Shigella* and increase that of *Bifidobacterium* and *Lactobacillus*, having a stronger regulatory effect on intestinal flora in humans [10]. The intestinal flora has been implicated in a variety of diseases, such as inflammatory bowel diseases and obesity [11]. Diabetes comprises a group of metabolic diseases characterized by hyperglycemia, characterized chronic hyperglycemia associated with long-term damage, dysfunction, and failure of different organs, especially the eyes, kidneys, nerves, heart, and blood vessels [12]. Currently, diabetes is becoming a global “epidemic” and is rapidly spreading worldwide [13]. According to the World Health Organization, 346 million people worldwide were diagnosed with diabetes ten years ago [14]. In the United States, diabetes mellitus (DM) was the seventh leading cause of death in 2019 [15]. Considering the rapid increase in the prevalence of DM, the development of hypoglycemic medicine and new antihyperglycemic technologies, especially for research on natural hypoglycemic substances for health purposes, is urgently needed. Aging is a natural, time-dependent physiological and complex process characterized by genomic instability, telomere attrition, epigenetic alterations, loss of proteostasis, deregulated nutrient sensing, mitochondrial dysfunction, cellular senescence, stem cell exhaustion, and altered intercellular communication, which leads to a gradual decrease in physiological fitness and a reduced ability to respond to environmental demands [16,17]. Age-related changes accelerate several age-associated cerebrovascular diseases, such as brain vessel dysfunction and neurodegenerative pathologies, among which Alzheimer’s disease is the most prominent [18]. Overall, antiaging studies with the aim of reducing aging-induced disease and increasing the health span are of crucial significance.

Although some clinical drugs are effective at preventing hypoglycemia and delaying senescence, they still have deficits in safety, especially for long-term use. As natural substances, many polysaccharides or their derivatives, such as ginseng polysaccharide [19], *Althea rosea* polysaccharide [20], the Momordica charantia L. polysaccharide–chromium (III) complex [21], Gomphus clavatus Gray polysaccharide [22], and exopolysaccharides from *Lactobacillus plantarum* HY7714 [23] and *Weissella confusa* KR780676 [24], exhibit both antihyperglycemic and antiaging activities. The zebrafish (Danio rerio) has emerged as a powerful vertebrate model system for the study of developmental processes, human diseases, and tissue regeneration [25]. In this study, an exopolysaccharide (Galacan) was extracted from a strain of *Agrobacterium* sp., which was obtained from the rhizosphere soil of chickpea in New Delhi, and it was studied for the first time. The total sugar content, FI-IR spectrum, monosaccharide composition, molecular weight, and residue linkages of Galacan were analyzed. Considering intestinal health, the regulation of Galacan on the proliferation of *Lactobacillus brevis* was first evaluated in zebrafish. Then, the antihyperglycemic and antiaging activities of Galacan were investigated in vivo in *Danio rerio*.

## 2. Materials and Methods

### 2.1. Materials

*Agrobacterium* sp. FN01 was isolated from the soil of western Sichuan Plateau and preserved in the China Center for Type Culture Collection (CCTCC, No. M2023226). The zebrafish were purchased from Hunter Biotechnology, Inc. (Hangzhou, China). *L. brevis* was purchased from the China General Microbiological Culture Collection Center (CGMCC 1.2028). Dextran standards were purchased from American Polymer Standards Corporation (APSC; Mentor, OH, USA). A galactose standard was purchased from Sinopharm (Beijing, China). The other chemicals used in this study were of analytical grade and obtained from Aobox (Beijing, China), Solarbio (Beijing, China), Merck (Darmstadt, Germany), Chron Chemicals (Chengdu, China), GHTECH (Guangdong, China), Aladdin (Shanghai, China), and Sinopharm (Beijing, China).

### 2.2. Preparation of Exopolysaccharides from Agrobacterium sp. FN01

*Agrobacterium* sp. FN01, stored at −80 °C, was grown in LB solid media at 30 °C for 24 h under aerobic conditions. A single colony was inoculated in 10 mL of LB broth and grown for 24 h at 30 °C. The seed solution was obtained after the bacterial suspension was transferred to 50 mL of fresh LB broth and cultured for 24 h at 30 °C. The seed solution (10^8^ CFU/mL) was inoculated in 5 L of fermentation medium (5.0% sucrose, 0.3% sodium nitrate, 0.2% peptone, 0.2% KH_2_PO_4_, 0.04% MgSO_4_, 0.002% FeSO_4_•7H_2_O, 0.0012% MnSO_4_, 0.01% CaCl_2_, and 0.2% defoamer (Zhuoyun Doubao, Changzhou, China)) and grown for 24 h at 30 °C [26]. Ethanol (95%) was added to the fermentation mixture at 3:1 (*v*:*v*) [27], and the mixture was kept at room temperature for 5 min. The crude exopolysaccharides were collected after filtration. The crude sample was resuspended in 5 L of distilled water and filtered using a diatomite filter aid. Then, 20 L of ethanol (95%) was added to the filtrate, and the mixture was kept at room temperature for 10 min. The exopolysaccharide was obtained after filtration and pressing to precipitate the product, which was named Galacan.

### 2.3. Total Sugar and Protein Content Determination

The phenol–sulfuric acid method was used to determine the total sugar content of Galacan, and the detailed protocol was as follows. Glucose was used as a standard. One milliliter of standard (0, 0.02, 0.04, 0.06, 0.08, or 0.1 mg/mL) and the dried Galacan (0.05 mg/mL) were mixed with 1 mL of 5% phenol in the tube. Sulfuric acid (5 mL) was added to the mixture, which was vortexed after 10 min. The reaction mixture was kept at 30 °C for 20 min, after which the absorbance at 490 nm was measured. A linear relationship was first established between the concentration of the standards and the OD490 (y = 0.1784x − 0.0133; R^2^ = 0.9977). The total sugar of Galacan was expressed as the glucose content after calculation according to the OD490 results.

The protein content was determined using the Coomassie brilliant blue method, and bovine serum albumin was used as a standard [28].

### 2.4. Fourier Transform Infrared Spectroscopy (FT-IR)

The FT-IR spectra were obtained over wavenumbers from 4000 cm^−1^ to 400 cm^−1^ using the KBr method [29].

### 2.5. Monosaccharide Composition, Molecular Weight (Mw), and Element Determination

The monosaccharide of Galacan was obtained after hydrolysis using sulfuric acid according to previous methods, with slight modifications [29]. First, Galacan (50 mg) was hydrolyzed with 5 mL of 1 M sulfuric acid in boiling water for 30 min. The mixture was then vortexed before being incubated in a 100 °C water bath for 2.5 h. The solution was neutralized with BaCO_3_ and concentrated by rotary evaporation. The sample was detected by HPLC. An evaporative light-scattering detector (ELSD) and a chromatographic column (Shodex HILIC pak VG-50 4E 250 mm × 4.6 mm, Shodex, Tokyo, Japan) were used for the determination. Acetonitrile and water (9:1) were used as the mobile phase. The column temperature was 40 °C, and the flow rate was 0.8 mL/min. A mixed standard of glucose and galactose (final concentration was 1 mg/mL) was prepared for quantitation.

The molecular weight of Galacan was also determined using an HPLC instrument equipped with an RID and a TSK gel GMPW XL column (300 mm × 7.8 mm, Tosoh Corp., Tokyo, Japan). The sample (5 mg/mL) was eluted with 0.05 M sodium nitrate supplemented with 0.05% sodium azide at 0.8 mL/min. Standards of dextran were also prepared (4300 Da, 37,600 Da, 216,400 Da, 1,194,000 Da, and 2,667,000 Da). The molecular weight of Galacan was calculated according to the linear relationship between retention time (RT) and logMw (y = 14.82 − 0.8826x, y: LogMw, x: RT; R^2^ = 0.9819).

Elements of carbon, nitrogen, and hydrogen were detected using a FlashSmart elementary analyzer (Thermo Scientific, Waltham, MA, USA). In addition to the contents of carbon, nitrogen, and hydrogen, an approximate content of oxygen could be obtained.

### 2.6. Periodate Oxidation, Smith Degradation, and NMR Analysis

#### 2.6.1. Periodate Oxidation

One hundred microliters of different concentrations of sodium periodate (NaIO_4_) solution (0.00, 3.75, 7.50, 11.25, 15.00, and 30.00 mmol/L) was added to 25 mL volumetric flasks filled with deionized water. The absorbance at 223 nm was measured. The linear relationship between OD223 and the concentration of NaIO_4_ was determined (y = 9.7427x − 0.0038, y: OD223, x: concentration of NaIO_4_). Galacan (24.7 mg) was dissolved in a NaIO_4_ (15 mmol/L) solution in a 25 mL volumetric flask and kept in the dark. One hundred microliters of the reaction mixture was added to another 25 mL volumetric flask at 0, 24, 48, 72, 96, 120, 144, 168, and 180 h, after which the OD223 was determined. Ethylene glycol was added to react with the residual NaIO_4_ in the reaction mixture when the absorbance was unchanged. According to the relationship between OD223 and NaIO_4_, the consumption of NaIO_4_ by Galacan was obtained. The production of formic acid in the reaction was measured using titration with 4 mg/mL of NaOH.

In addition to NaIO_4_ and formic acid, the other reaction solutions were dialyzed and freeze-dried for salt degradation.

#### 2.6.2. Smith Degradation

The lyophilized powder was dissolved in 4 mL of 2 mol/L trifluoroacetic acid (TFA), hydrolyzed at 120 °C for 2 h, and dried using a rotary evaporator at 43 °C by adding methanol several times to remove TFA. The powder was reacted with 2 mL of 4% NaBH_4_ and neutralized with 25% acetic acid. The boronic acid was also removed using a rotary evaporator by adding methanol several times at 43 °C. The reaction product was vacuum-dried at 55 °C for 2 h and then acetylated by adding 2 mL of anhydrous pyridine and 2 mL of acetic anhydride and reacted at 70 °C for 4 h. After the pyridine and acetic anhydride were evaporated by adding methylbenzene, the product was resuspended in trichloromethane and dried again using a rotary evaporator at 43 °C by adding methanol several times. The degree of acetylation was determined using a gas chromatography–mass spectrometry (GC–MS) instrument equipped with a DB-WAX column (30 m × 0.25 mm × 0.25 μm). The temperature of the injector port was 270 °C. The reaction started at 120 °C, followed by a 2 °C/min gradient to 200 °C and then to 270 °C at a 5 °C/min gradient. The carrier gas was helium, and the flow rate was 1 mL/min. The mass spectrometer was operated in electron impact mode at 70 eV, and the ion source temperature was 220 °C.

#### 2.6.3. NMR Analysis

Due to the large high molecular weight of Galacan, NMR analysis necessitated its prior degradation. Initially, a 2% solution of Galacan underwent hydrolysis with 1% sulfuric acid at 90 °C for 30 min. The resulting hydrolysates were subsequently neutralized with a sodium hydroxide (NaOH) solution, followed by ultrafiltration and concentration. To isolate low-molecular-weight components of Galacan (referred to as LG), five times the volume of 95% ethanol was added to the solution. The resulting sediments, representing the low-molecular-weight fraction of Galacan, were collected, dried, and dissolved in deuterated water (D_2_O) within an NMR tube. Subsequently, a comprehensive array of NMR spectroscopic techniques, including ^1^H NMR, ^13^C NMR, distortionless enhancement by polarization transfer homonuclear-135° (DEPT135), ^1^H/^1^H correlation spectroscopy (COSY), heteronuclear single-quantum coherence (HSQC), and heteronuclear multiple-bond correlation (HMBC), were conducted. These analyses were performed using a Bruker Avance 500 MHz NMR spectrometer (Brucker Co., Rheinstetten, Germany).

### 2.7. Regulation of Galacan on the Growth of L. brevis in Intestinal Flora of Zebrafish

Previous studies have indicated that *L. brevis* exhibited various functional activities, such as enhancing oxidative stress resistance, extending the lifespan, improving blood glucose and insulin resistance, and ameliorating other physiological aspects [30,31]. In this study, the effect of Galacan on the growth of *L. brevis* was further evaluated. *L. brevis* (CGMCC 1.2028) was first labeled by Vybrant™ CM-Dilcell-labeling solution (CM-DiI) (ThermoFisher Scientific, Waltham, MA, USA). AB wild-type zebrafish (5 dpf) were purchased from Hunter Biotechnology, Inc. (Hangzhou, China). A total of 150 fish were fed with 1 × 10^8^ CFU/mL of labeled *L. brevis* at 28 ± 2 °C until 6 dpf and then assigned to 5 groups in 6-well plates (30 fish/group, 3 mL/well). The five groups were the model control (without other treatment, mCTRL), position control (fish induced with 2000 μg/mL of live combined *Bacillus subtilis* and enterococcus faecium granules (Hanmi Pharm, Beijing, China), POS), and Galacan (250, 500, and 1000 μg/mL)-treated groups (Galacan-treated group, GAL). After 6 h, 10 fish were randomly chosen to be observed under fluorescence microscope (AZ100, Nikon, Tokyo, Japan). The fluorescence intensity was analyzed using NIS-Elements D 3.20.

All procedures involving animals were conducted with the approval of the Experimental Animal Ethics Committee of the Feed Research Institute of the Chinese Academy of Agricultural Sciences (Beijing, China), and they adhered strictly to the regulations and guidelines set forth by the same committee. The ethical approval number for this study was IFR-CAAS20221115.

### 2.8. Antihyperglycemic Evaluation of Galacan in Zebrafish

AB wild-type zebrafish (3 days post-fertilization, 3 dpf) were purchased from Hunter Biotechnology, Inc. (Hangzhou, China). A total of 180 fish were assigned to 5 groups in beakers (30 fish/group; 15 male and 15 female fish). The six groups were the blank control (without treatment, CTRL), diabetes model control (fish induced with STZ), positive control (metformin-treated diabetes group, STZ + MTF), and Galacan-treated (62.5, 125, and 250 μg/mL) groups (Galacan-treated diabetes group, STZ + GAL). Metformin (400 μg/mL) and Galacan (62.5, 125, and 250 μg/mL) were added to the water. To develop a diabetic zebrafish model, the fish were injected with streptozotocin through the yolk sac, which provided 0.15% egg yolk powder solution during the day and 3% glucose solution at night by dissolving in water. The solution for each group was replaced daily. After diabetes was induced in the zebrafish for 2 days at 28 ± 2 °C, the fish were washed 3 times with standard diluted water and dried. Blood glucose was measured by a glucometer (ACCU-CHEK Performa, Roche Diagnostics, Shanghai, China). A decrease in blood glucose suggested antidiabetic capacity.

Furthermore, mutant zebrafish (3 dpf) with pancreatic β cells expressing green fluorescence were obtained from Hunter Biotechnology, Inc. (Hangzhou, China), treated again according to the above method, and observed after 2 days of treatment by fluorescence microscopy. The fluorescence intensity was analyzed using NIS-Elements D 3.20. The fluorescence intensity indicated the degree of damage to the pancreatic island.

### 2.9. Antiaging Effect of Galacan on Zebrafish

AB wild-type zebrafish embryos (6 h post-fertilization, 6 hpf) were randomly distributed in 6-well plates (30 fish/well; 15 male and 15 female fish). A blank control (without treatment, CTRL) and aging model zebrafish (treated with hydrogen peroxide (HP), 1000 μM) were used. Catalase (2000 μg/mL) was used as a positive control (HP + CAT) for comparison with the results of the Galacan-treated groups (HP + GAL) at different concentrations of 125, 250, and 500 μg/mL. Galacan was also supplied by dissolving in water. The solution for each group was replaced daily. The experiments were performed for 6 days at 28 ± 2 °C. The level of senescence-associated β-galactosidase (SA-β-gal) was measured using a staining kit (Beyotime, Shanghai, China). Ten zebrafish embryos were randomly selected for observation by fluorescence microscopy (SZX7; Olympus, Tokyo, Japan) and analyzed through NIS-Elements D 3.20. The increase in fluorescence intensity suggested that Galacan could inhibit β-galactosidase activity.

Telomerase activity in the cells of zebrafish embryos (6 hpf) was also detected after treatment with catalase and Galacan. The treatments were the same as those described for SA-β-gal. After induction for 6 days at 28 ± 2 °C, telomerase activity was measured via a Zebrafish TE Elisa Kit (Hengyuan Biology Science and Technology Co., Ltd., Shanghai, China). The results were used to analyze the effect of Galacan on the activity of telomerase in zebrafish embryos.

### 2.10. Statistical Analysis

Results of histograms were expressed as the mean ± SD. The experiments were repeated at least in triplicate. Statistical significance was analyzed by one-way ANOVA using SPSS 26.0 software. Values of *p* < 0.05 were considered statistically significant.

## 3. Results and Discussion

### 3.1. Total Sugar and Protein Content of Galacan

The total sugar content of Galacan was measured by the phenol–sulfuric acid method, and the results showed that the total sugar content was 96.44 ± 1.09%. The protein concentration in Galacan was 0.81 ± 0.09%. Natural polysaccharides generally coexist with proteins, nucleic acids, lipids, and amino acid residues, which are linked by either hydrophobic interactions or hydrogen bonding, cavities, or crevasses [32]. Therefore, the EPSs extracted from bacteria always contain small amounts of proteins. Similarly, a previous study reported the presence of trace levels of protein (0.45%) in polysaccharides derived from *Lactobacillales*, with 99.09% total sugar [33]. A recent report also shows that an exopolysaccharide extracted from a halotolerant cyanobacterium contained 0.07% proteins [34].

### 3.2. FT-IR Analysis

**Figure 1:**
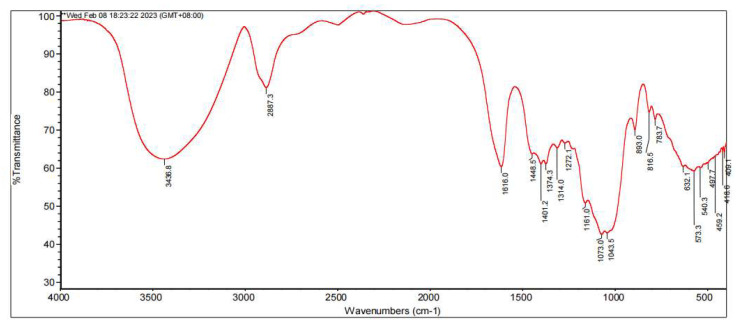
FT-IR spectrum of Galacan.

FT-IR spectroscopy is an important tool useful for qualitatively investigating biopolymers. The FT-IR spectrogram of Galacan is presented in Figure 1. Many complex peak patterns, including characteristic absorption peaks of polysaccharides, appeared from 3000^−1^ to 1000 cm^−1^ in the spectrum. As reported, carbohydrates are generally recognized by absorption peaks at 3400 cm^−1^ and 2940 cm^−1^, which are the –OH stretch peak and C–H stretch peak, respectively [35]. The Galacan exhibited a broad stretching hydroxyl group at 3436.8 cm^−1^ and a weak C-H stretching vibration at 2887.3 cm^−1^, indicating polysaccharide characteristics. The absence of a peak between 1700 and 1775 cm^−1^ suggested that there was no glucuronic acid or diacetyl ester in Galacan [36,37]. The strong absorption peak at 1616.0 cm^−1^ corresponded to the amide >C=O stretch, and the peak from 1314.0 to 1448.5 cm^−1^ was assigned to the >C=O stretch of the COO- groups and the C-O bond from COO- [27,38]. The broad stretching of C–O–C and C–O at 1000–1200 cm^−1^ suggested the presence of carbohydrates [27,35,39]. Therefore, the strongest absorption bands in the fingerprint region (region below 1500 cm^−1^ where bands characterize the molecule as a whole [35]) at 1073.0 cm^−1^ and 1043.5 cm^−1^ were attributed to polysaccharide. The absorption at 893.0 cm^−1^ indicated the β-configuration, whereas no α-configuration was recorded at approximately 840 cm^−1^ [5,40]. The characteristic absorption peaks of carboxyl and hydroxyl groups present in Galacan are similar to those of other reported exopolysaccharides from other bacteria, such as *Weissella cibaria* [33] and *Lactobacillus casei* LC2W [41]. All of them included a broad peak of absorption between 3700 and 3000 cm^−1^ (–OH), an absorbance around 2900 cm^−1^ (C–H), and a peak around 1600 cm^−1^ (COO−).

### 3.3. Results of Monosaccharide Composition, Mw, and Elemental Analysis of Galacan

**Figure 2:**
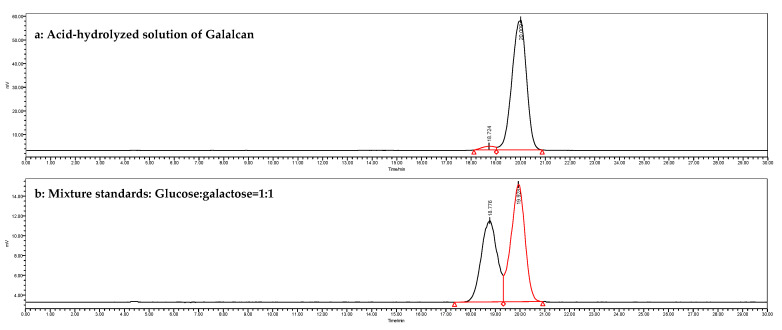
HPLC chromatograms of monosaccharide determination. (**a**) Chromatogram of an acid-hydrolyzed solution of Galalcan and (**b**) mixture of standards (glucose:galactose = 1:1).

Figure 2 shows that, compared with the chromatograms of mixed standards, the spectrum of Galacan exhibited two apparent peaks at 18.776 and 19.928 min, corresponding to galactose and glucose, respectively. Galacan was only composed of glucose and galactose at a molar ratio of 25:1. The presence of different monosaccharides suggested that Galacan is a heteropolysaccharide. Similarly, exopolysaccharides composed of only glucose and galactose were also found in other previous studies [33,35].

**Figure 3:**
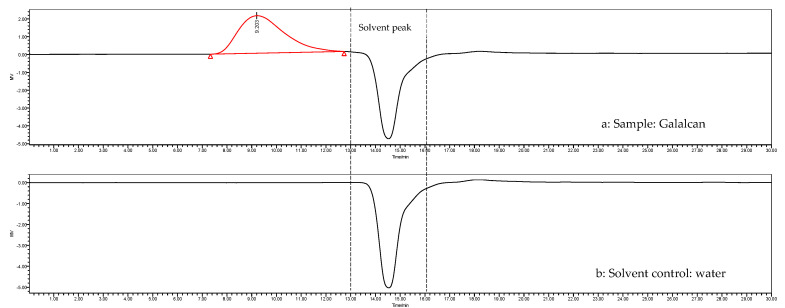
HPLC chromatogram of a TSK gel GMPWXL column eluted with 0.05 M NaNO_3_ supplemented with 0.05% sodium azide. (**a**) Chromatogram of Galacan solution and (**b**) chromatogram of solvent (water).

As shown in Figure 3, the sample only had a single peak in the HPLC elution profile. According to the linear relationship of dextran, the molecular weight (Mw) of Galacan was determined to be 4982.327 kDa. Earlier studies have reported that the molecular masses of most heteropolysaccharides obtained from lactic acid bacteria are between 1 × 10^4^ Da and 6 × 10^6^ Da [42,43,44]. The molecular weight of Galacan is approximately the upper limit of some exopolysaccharides from *Lactobacillus*, which are high-molecular-weight polymers. Galacan solution is highly viscous and even difficult to dissolve at high concentrations, possibly because of its large molecular weight. The Mw of polysaccharides is intimately linked to their solubility, viscosity, rheological behavior, and numerous other physicochemical properties. Galacan exhibits a notably high Mw, which should be paid more attention in the further applications.

The element results showed that Galacan contained 41.08% carbon, 6.04% hydrogen, and less than 0.3% nitrogen. These data suggest that there is little nitrogen in Galacan. Based on the total sugar content, FI-IR spectrum, and monosaccharide composition, it is reasonable to speculate that Galacan is a polysaccharide that contains mostly carbon, hydrogen, and oxygen. The oxygen content in Galacan was approximately 52.58%. The molar ratio of carbon, hydrogen, and oxygen was inferred to be 1.04:1.84:1.00.

### 3.4. Structural Characterization of Galacan

#### 3.4.1. Analysis of Periodate Oxidation and Smith Degradation

**Figure 4:**
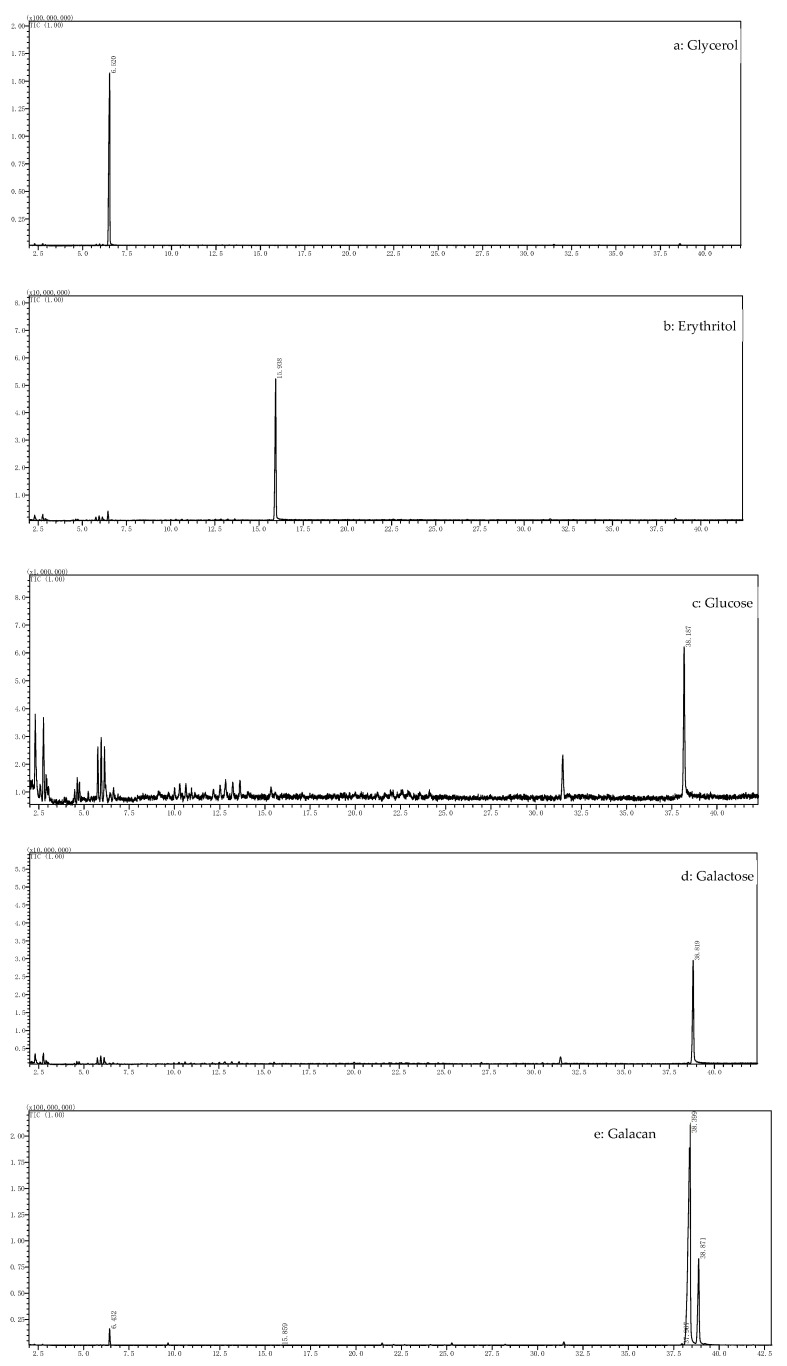
GC chromatograms of the Smith degradation product of Galacan and acetylized standards. (**a**–**d**) Acetylation product chromatograms of glycerol, erythritol, glucose and galactose. (**e**) Chromatogram of the Smith degradation product.

After 180 h, the absorbance (OD223) of the periodate oxidation solution was maintained at 0.490 (Table 1). During the reaction, 0.0582 mmol of NaIO_4_ was consumed by 24.7 mg of Galacan, and 0.0215 mmol of formic acid was produced (Table 2). The molar ratio of Galacan to NaIO_4_ consumption to formic acid production was 1:0.3819:0.1411, suggesting that there were 14.11% (1→) or (1→6) linkages in Galacan [45]. NaIO_4_ consumption was more than twice that of the formic acid product, indicating the presence of monosaccharides that were (1→2), (1→2,6), (1→4), or (1→4,6) linked (9.97%). The periodate-oxidized product was then fully hydrolyzed and detected by GC, and the spectrograms are shown in Figure 4. The results showed that the alditol acetates of polyalcohols could be oxidized to produce alditol acetates of glycerol and slight erythritol alditol acetates, suggesting the presence of a few (1→2) or (1→6) linkages and little or no (1→4) or (1→4,6) linkages. GC results also demonstrated that glucose and galactose were produced after periodate oxidation, indicating that glycosidic linkages cannot be oxidized by NaIO_4_, which suggested that (1→3), (1→3,6), (1→2,3), (1→2,4), (1→3,4), or (1→2,3,4) linkages might be present in the structure of Galacan [45,46].

#### 3.4.2. NMR Results

**Figure 5:**
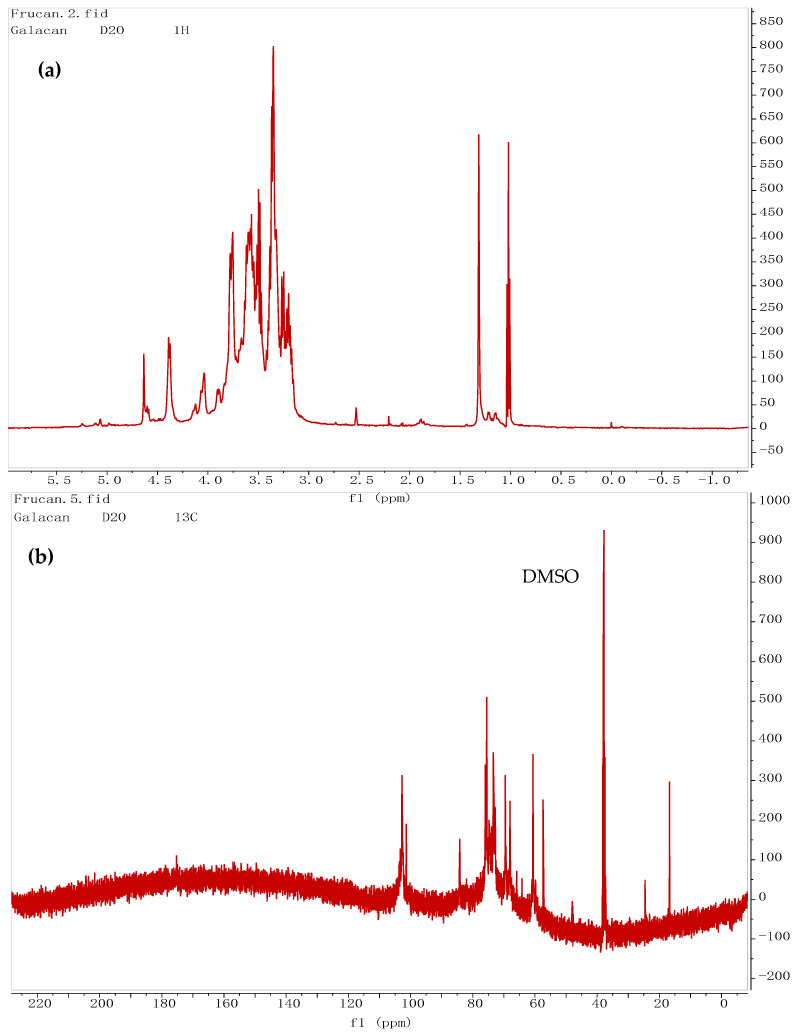

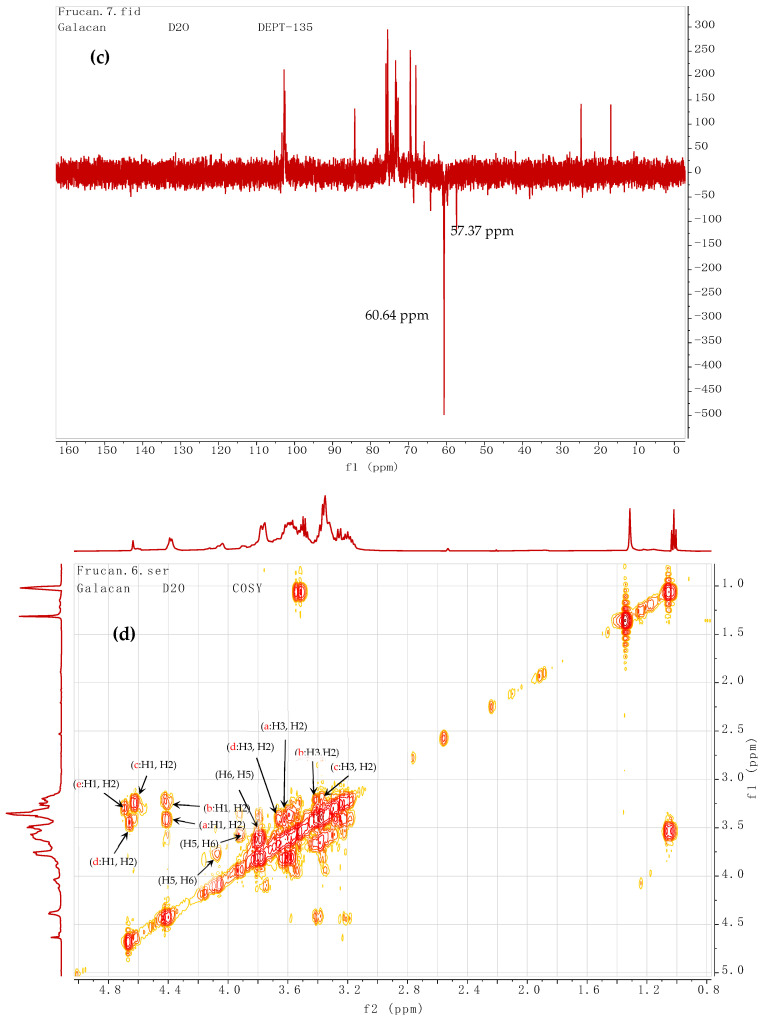

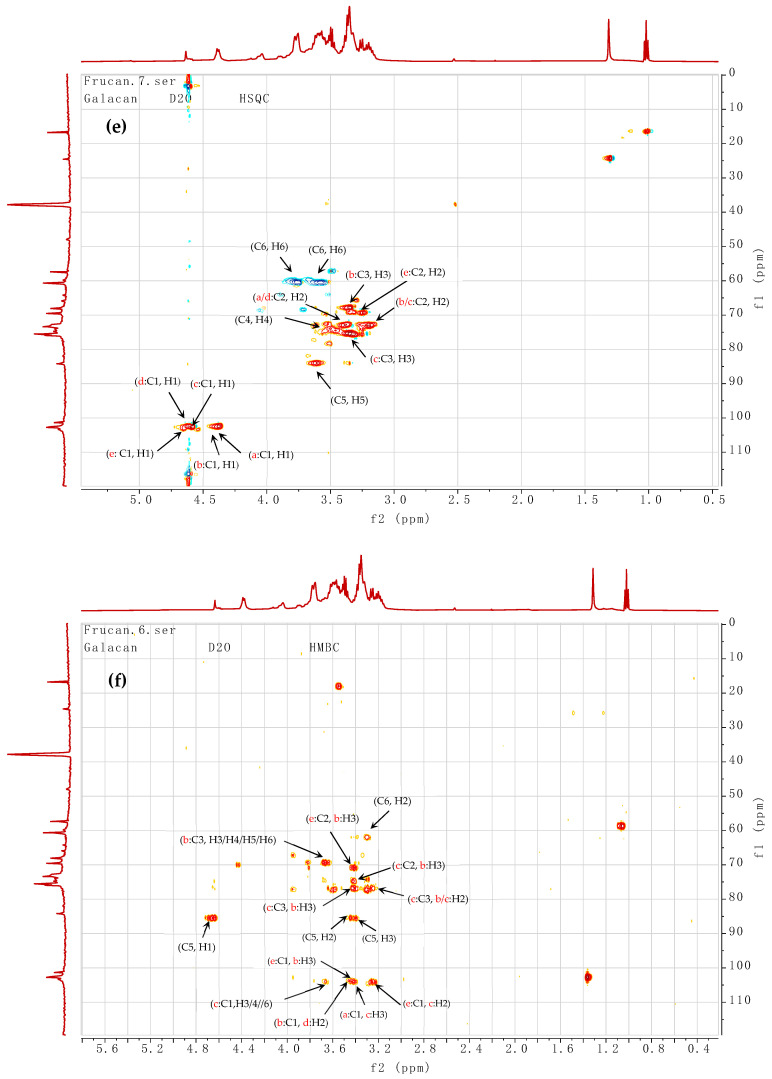

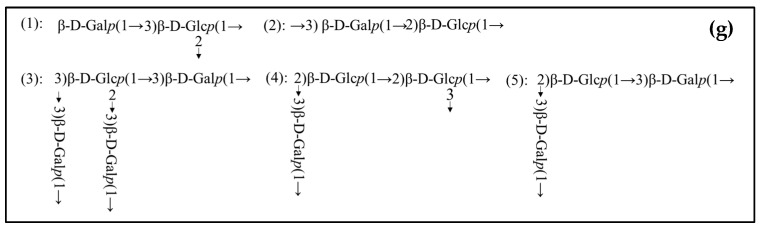
The ^1^H NMR (**a**), ^13^C NMR (**b**), DEPT (**c**), ^1^H–^1^H COSY (**d**), HSQC (**e**), and HMBC (**f**) spectra of low-molecular-weight Galacan (LG) and possible structure in Galacan (**g**).

The structure of Galacan was further elucidated through NMR analysis using the LG sample. In the ^1^H NMR, the β-anomeric protons typically manifest in the δH 3–5 ppm range, while α-anomeric protons are commonly observed in the δH 5–6 ppm range [47]. The ^1^H NMR spectrum of LG depicted anomeric signals at 4.38, 4.39, 4.58, 4.60, and 4.64 ppm, attributed to protons of the β-glycosidic bond (Figure 5a). These signals, denoted as a, b, c, d, and e, respectively, demonstrated an increasing order of chemical shifts. Specifically, the anomeric proton signals at 4.58, 4.60, and 4.64 ppm were associated with glucose residues, while those at 4.38 and 4.39 ppm were attributed to galactose residues, partly based on the monosaccharide composition. Moreover, proton signals in the region of 4.3–3.0 ppm were assigned to H2–H6 of the sugar residues [48]. In the ^13^C NMR spectrum of LG (Figure 5b), five anomeric carbon signals were observed at 102.66, 102.53, 102.35, 102.77, and 101.37 ppm, indicating the presence of various sugar residues.

The anomeric carbon peaks between 60 and 90 ppm were attributed to C2–C6 [47]. Further analysis via the DEPT spectrum (Figure 5c) revealed the signals at 60.64 and 57.37 ppm were attributed to the C-6 non-substituted glycosidic residues [48]. All the ^1^H and ^13^C signals were assigned based on COSY (Figure 5d), HMQC (Figure 5e), and HMBC (Figure 5f), with a comprehensive summary provided in Table 3.

The COSY spectrum (Figure 5d) displayed H1–H2 correlations of the sugar residues, with signals between 3.80 ppm and 3.2 ppm overlapping heavily, making it challenging to assign H3, H4, H5, and H6. In the HMQC spectrum (Figure 5e), direct C–H couplings were evident. The anomeric proton signals at 4.38, 4.39, 4.58, 4.60, and 4.64 ppm were related to 102.66, 102.35, 102.53, 101.37, and 102.77 ppm, respectively, which were the residues of a, b, c, d, and e. Combined with the COSY spectrum, the signals at 72.85, 73.23, 73.38, 72.92, and 69.50 ppm were attributed to the C2 of a, b, c, d, and e, respectively. The proton signals at 68.00 and 75.50 ppm were attributed to the C3 of b and c, respectively. Based on the analysis of the HMBC spectrum (Figure 5f), the C1 at 102.66 ppm of a was correlated to the H3 signal at 3.32 ppm of c. The C1 at 102.35 ppm of b was correlated to the H2 signal at 3.39 ppm of d. The C1, C2, and C3 signals at 102.53, 73.38, and 75.50 ppm of c were all correlated to the H3 signal at 3.35 ppm of b. The C1 signal at 102.77 ppm of e was related to the H2 signal at 3.21 ppm of c and H3 signal at 3.35 ppm of b. Moreover, the C2 signal at 69.50 ppm of e was also correlated to the H3 signal at 3.35 ppm of b. Overall, the residues of a, b, c, d, and e were determined to be β-D-Galp(→, →3)β-D-Galp(1→, →2,3)β-D-Glcp(1→, β-D-Glcp(1→, and →2)β-D-Glcp(1→. Through the combined analysis of periodate oxidation, Smith degradation, and NMR, five potential structures of fragments within Galacan were proposed, as depicted in Figure 5g. Polysaccharides are generally complexed structures, especially for heteropolysaccharides. As Galacan exhibited high viscosity, LG was used for part structure analysis. However, there are also numerous superimposed signals in ^1^H NMR and ^13^C NMR spectrums, limiting the amount of information that can be directly extracted. This phenomenon suggests that Galacan possesses a highly complex molecular architecture. Nevertheless, we are optimistic that our study will serve as a helpful foundation for future research endeavors aimed at unraveling the intricate details of this polysaccharide.

### 3.5. Effect of Galacan on the Growth of L. brevis

**Figure 6:**
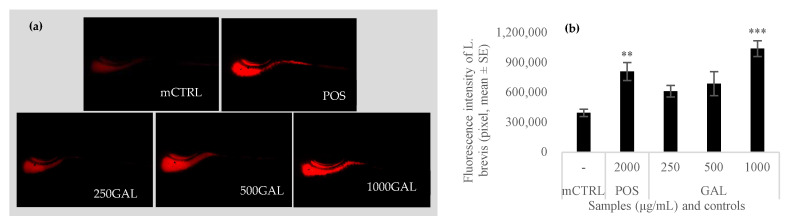
Fluorescence photographs (**a**) and intensity (**b**) of labeled *L. brevis* in zebrafish untreated and treated with different concentrations of Galacan (*n* = 10). mCTRL: fish only pretreated with *L. brevis*; POS: pretreated fish were fed with live combined *Bacillus subtilis* and *Enterococcus faecium* granules; GAL: pretreated fish were fed with Galacan. Significant differences between the mCTRL group and the other groups were detected (** *p* < 0.01, *** *p* < 0.001).

In *L. brevis*-pretreated zebrafish, Galacan enhanced the growth of *L. brevis* in the intestinal tract (Figure 6). The fluorescence-labeled *L. brevis* were first observed by fluorescence microscope, and the results are shown in Figure 6a. The fish of the *L. brevis* model had dark bodies. The fish of the positive group showed obvious red fluorescence. After being induced with Galacan at 250, 500, and 1000 μg/mL, the fluorescence in zebrafish was dose-dependently enhanced. The fluorescence intensity was also calculated (Figure 6b) and the results were consistent with the photographs. The intensity of fish in mCTRL was only 395,929 ± 36,791. Compared to the positive control (809,397 ± 90,617), Galacan showed no obvious effect at 250 μg/mL (611,879 ± 57,981) and 500 μg/mL (688,166 ± 120,744). However, the fluorescence intensity in fish was significantly improved after treatment with 1000 μg/mL of Galacan (1,039,653 ± 78,889; *p* < 0.001). Both qualitative and quantitative analysis suggested that Galacan was effective in the growth promotion of *L. brevis* in zebrafish.

Probiotics have a positive effect on the health of the host [49], including immunoregulation, protection against infections, relief of irritable bowel symptoms, decrease in the gut inflammatory response, prevention of allergies, and others [50]. Many probiotic bacteria are members of the intestinal microbiota and play an important role in intestinal health. As an important probiotic, *L. brevis* widely exists in the intestinal tract of animals and has many biologic activities. It has been reported that *L. brevis* (AY858) can stabilize the intestinal microbiota and inhibit the growth of pathogenic bacteria, having significant effects on protecting mice from the gut inflammation induced by ETEC [9]. Furthermore, metabolites of *L. brevis*, such as polyphosphate, were also effective in the modification of the intestinal microbiome and enhancement of the intestinal barrier integrity [51]. Kochetkov also studied the positive effect of *L. brevis* (47f) in zebrafish and suggested that *L. brevis* 47f could mitigate the toxic effects of sublethal concentrations of imidacloprid on *Danio rerio* [52]. In our study, Galacan improved the content of *L. brevis* in zebrafish, which was significantly important for the health of the host.

### 3.6. Antihyperglycemic Activity of Galacan

**Figure 7:**
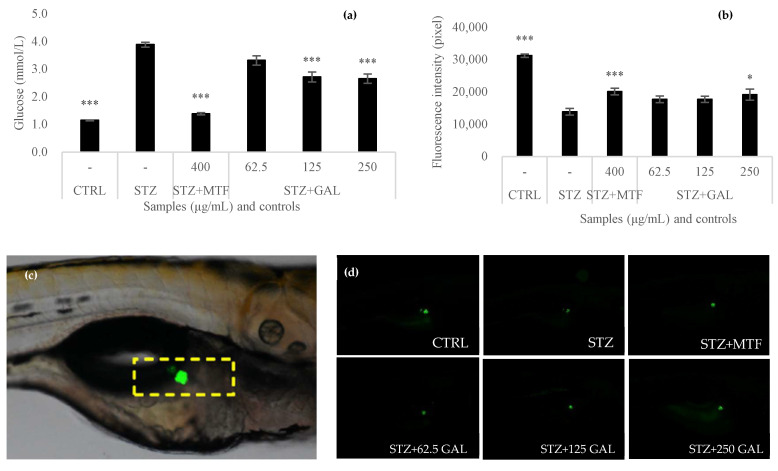
Antihyperglycemic effects of Galacan evaluated in zebrafish. (**a**) Blood glucose levels after the zebrafish model of diabetes was established by treatment with metformin or different concentrations of Galacan (*n* = 10). (**b**) Fluorescence intensity of pancreatic β cells in zebrafish after treatment with metformin or different concentrations of Galacan (*n* = 10). (**c**,**d**) Fluorescence observation of different zebrafish groups by fluorescence microscopy. CTRL: blank control; STZ: diabetes model control induced with streptozotocin; STZ + MTF: positive control of diabetes model treated with metformin; STZ + GAL: diabetes model treated with Galacan (62.5, 125, and 250 μg/mL). Significant differences between the STZ group and the other groups were detected (* *p* < 0.05 and *** *p* < 0.001).

In this work, we evaluated the antihyperglycemic activity of three doses of Galacan compared with that of metformin in streptozotocin-induced hyperglycemic zebrafish. As shown in Figure 7a, the blood glucose level was maintained at 1.15 ± 0.017 mmol in the CTRL group, while it increased to 3.89 ± 0.087 mmol in the STZ group. Metformin, used as a positive control, significantly decreased blood glucose to 1.39 ± 0.038 mmol (*p* < 0.001) compared to that in the STZ group. Galacan also exhibited antihyperglycemic effects, especially at 125 and 250 μg/mL of Galacan. In the STZ + GAL group, the blood glucose concentration was 3.32 ± 0.171 mmol when the concentration of Galacan was 62.5 μg/mL, which was slightly different from that in the STZ group. However, it significantly decreased to 2.72 ± 0.181 and 2.66 ± 0.167 mmol at 125 and 250 μg/mL, respectively.

Additionally, pancreatic β cells from zebrafish were also evaluated to determine the ability of Galacan to protect pancreatic β cells from damage. As shown in Figure 7c,d, the fluorescence in the STZ group was significantly lower than that in the control group. After the addition of MTF and GAL, the fluorescence slightly increased. Furthermore, the results of the quantitative determination (Figure 7b) were generally consistent with the observations by fluorescence microscopy. Although the fluorescence intensity in the STZ + GAL group was less than that in the STZ + MTF group, it was significantly greater than that in the STZ group, especially at a concentration of 250 μg/mL (*p* < 0.05). Galacan was effective at protecting pancreatic β cells, suggesting a positive contribution to the antihyperglycemic mechanism.

DM was classified into type 1 DM (DM1) and type 2 DM (DM2) by the WHO (1999, with amendments), with type 1 DM (DM1) resulting from destruction of pancreatic β-cells, usually leading to absolute insulin insufficiency, and type 2 (DM2) mainly related to insulin resistance and relative insulin insufficiency [13]. Both DM1 and DM2 are closely related to the function of pancreatic islets. When the islet is destroyed, the blood glucose level in the body cannot be balanced by enough insulin, which leads to hyperglycemia. As in humans, blood glucose in zebrafish is also regulated by fasting or feeding [53]. Intraperitoneal injection of streptozotocin (STZ) into adult wild-type zebrafish resulted in a sustained hyperglycemic state, as determined by elevated fasting blood glucose values and increased glycation of serum protein, and these findings can be used as a zebrafish model of type I diabetes [25]. In the present study, a diabetic zebrafish model was induced by STZ. Both the glucose level and fluorescence results suggested that Galacan protected against hyperglycemia by protecting zebrafish against damage caused by STZ. The fluorescence intensity of pancreatic β cells in zebrafish indicated that a high concentration of Galacan significantly improved the viability of pancreatic β cells. Loss of functional insulin-producing β cells is a central diabetes mechanism for both type 1 and type 2 diabetes [15]. Thus, Galacan may have antidiabetic activity by protecting pancreatic β cells. Recently, pancreatic β-cell rescue and replacement therapies have become promising therapeutic avenues for combatting diabetes mellitus [15]. Galacan may be a promising active substance for rescuing pancreatic β cells and reducing diabetes incidence in the future.

### 3.7. Antiaging Activity of Galacan

**Figure 8:**
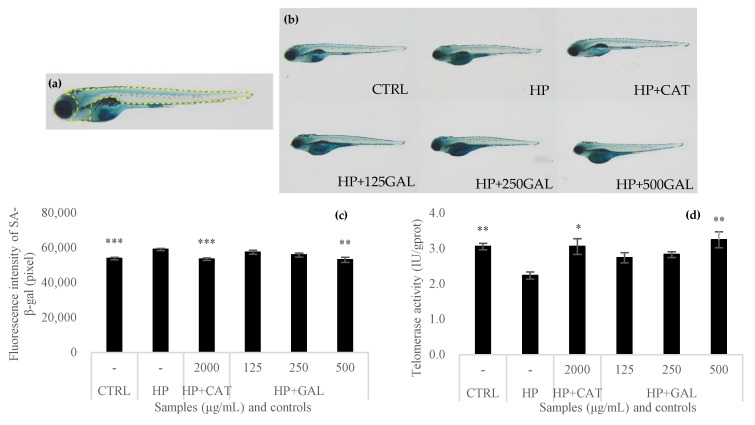
Antiaging effects of Galacan on zebrafish. (**a**,**b**) Fluorescence observation of different zebrafish groups by fluorescence microscopy. (**c**) Fluorescence intensity of senescence-associated β-galactosidase (SA-β-gal) after aging model zebrafish were treated with catalase and different concentrations of Galacan (*n* = 10). (**d**) Telomerase activity in cells of zebrafish embryos after treatment with catalase and Galacan (*n* = 3). CTRL: blank control; HP: aging model control induced with hydrogen peroxide; HP + CAT: positive control of aging model treated with catalase; HP + GAL: aging model treated with Galacan (125, 250, and 500 μg/mL). Significant differences between the HP group and the other groups were detected (* *p* < 0.05, ** *p* < 0.01, and *** *p* < 0.001).

As shown in Figure 8a, a normal young zebrafish is thin, has a light body color, and has a clear stripe. Zebrafish treated with different inducers were first observed by fluorescence microscopy and are shown in Figure 8b. The aging model fish treated with catalase (HP + CAT) were similar to the untreated fish. The fish induced with only hydrogen peroxide (HP) were obese, had a darker body color, were blurry, and exhibited obvious thickening. After Galacan was added, the body state of the model fish (HP + GAL) improved. In particular, the fish were thinner and clearer at 500 μg/mL. In addition, the fluorescence intensity was also measured, and the results are shown in Figure 8c. The fluorescence intensity in the HP + CAT group (53,854 ± 706 pixels) was almost the same as that in the CTRL group (53,500 ± 629 pixels) and was significantly different from that in the HP group (59,073 ± 629 pixels; *p* < 0.001). For the HP + GAL group, the fluorescence intensity significantly decreased to 53,114 ± 1408 pixels when the concentration of Galacan was 500 μg/mL (*p* < 0.01). Both qualitative observation and fluorescence analysis suggested that Galacan is effective at preventing aging.

A shortened telomere length contributes to cell death, generating body senescence. Telomerase can maintain the length of telomeres, and its activity was analyzed in the present work (Figure 8d). As a positive control, the addition of catalase increased telomerase activity from 2.24 ± 0.098 IU/gprot (HP group) to 3.06 ± 0.222 IU/gprot (HP + CAT group). Galacan improved telomerase activity in a concentration-dependent manner. The activity of telomerase was 2.74 ± 0.145 and 2.83 ± 0.084 IU/gprot at concentrations of 125 and 250 μg/mL, respectively. Even greater activity (3.25 ± 0.228 IU/gprot) was detected at 500 μg/mL than in the CTRL group (3.06 ± 0.091 IU/gprot). Galacan has been shown to have a protective effect on telomerase activity, demonstrating that it is an effective antiaging substance.

The mechanism of senescence is generally divided into two theories, stochastic and developmental genetic, the latter of which is familiar to the public and genetically predetermined as part of the continuum of birth, growth, maturation, and death [16]. Among the models of human senescence, zebrafish (*Danio rerio*) has been used as a highly promising model for studies of vertebrate senescence [54]. During early embryonic zebrafish development, β-galactosidase is a senescence-associated biomarker that is predictive of the premature aging phenotype [18]. In addition, telomere loss is thought to control entry into senescence [55], and cells that express telomerase could prevent telomere shortening and have extended lifespans [16]. In recent years, delaying senescence and preventing geriatrics have become a focus of research both domestically and internationally. The development of effective antiaging drugs or functional foods is an important research direction. A plethora of natural compounds, including quercetin, luteolin, catechins, resveratrol, curcumin, and lignans, have been reported to show antiaging activities. Among natural products, polysaccharides stand out as one of the esteemed natural products that also demonstrate notable antiaging properties. The results of the present study suggested that the exopolysaccharide from *Agrobacterium* sp. FN01 (Galacan) has an antiaging ability by restraining the activity of β-galactosidase and enhancing the activity of telomerase. Although Galacan has never been reported previously, several other polysaccharides from various materials exhibiting antiaging activity have been studied. The antiaging effects of the polysaccharide TLH-3 from *Tricholoma lobayense* and the exopolysaccharides from *Agrocybe cylindracea* were evaluated in vivo in a D-galactose-induced aging mouse model. The activity of superoxide dismutase (SOD) and CAT in the liver homogenate and serum of the TLH-treated model mice was noticeably increased, and the content of malonaldehyde (MDA) was significantly decreased in the liver and serum of the D-galactose-induced aging mice, compared with those in the model group (*p* < 0.05), alleviating oxidative damage in the serum and liver tissue of the D-gal-induced aging mice [56]. The exopolysaccharide extract of Agrocybe cylindracea also exhibited potential antiaging effects through decreasing MDA and total cholesterol (TC) levels, as well as increasing SOD and glutathione peroxidase (GSH-Px) activities and total antioxidant capacity (T-AOC) [57]. Therefore, natural polysaccharides are promising antiaging agents that deserve extensive and thorough research and are important for both drug development and functional food research.

## 4. Conclusions

The exopolysaccharide from *Agrobacterium* sp. FN01 (Galacan) is a heteropolysaccharide primarily composing glucose and galactose. Structural analysis revealed the presence of sugar residues, including β-D-Galp(→, →3)β-D-Galp(1→, →2,3)β-D-Glcp(1→, β-D-Glcp(1→, and →2)β-D-Glcp(1→. In vivo assessment using a zebrafish model demonstrated the multifaceted benefits of Galacan. It notably stimulated the proliferation of *L. brevis* within the intestine, showing its potential probiotic-enhancing properties. Additionally, Galacan exhibited significant antihyperglycemic effects by effectively lowering blood glucose levels and enhancing the fluorescence intensity of pancreatic β cells. Furthermore, its antiaging properties were evident through the inhibition of β-galactosidase activity and the enhancement of telomerase activity. All the results showed the practical significance of exopolysaccharides from *Agrobacterium* sp. FN01 in both the food and pharmaceutical industries. The versatile biological activities exhibited by Galacan highlight its potential as a valuable ingredient for product development in various applications.

## Figures and Tables

**Table 1:** The absorbance variability of sodium periodate (NaIO_4_) solution at different times after reacting with Galacan.

Time (h)	0	24	48	72	96	120	144	168	180	192
OD223	0.567	0.543	0.533	0.526	0.520	0.510	0.506	0.496	0.490	0.490

**Table 2:** Results of NaIO_4_ consumption, formic acid production, and related index calculation.

Sample	Weight (mg)	NaIO_4_ Consumption (mmol)	Formic Acid Production (mmol)	NaIO_4_ Consumption/ Formic Acid Production	NaIO_4_ Consumption/ Hexose Value
Galacan	24.7	0.0582	0.0215	2.7068	0.3819

**Table 3:** Chemical shift assignment of glycosidic linkages of low-molecular-weight Galacan (LG) in D_2_O.

Glycosidic Linkages	Number of ^13^C	^13^C δ(ppm)	HSQC (^13^C × ^1^H)	COSY (^1^H × ^1^H)	HMBC (^13^C→^1^H)
a: β-D-Gal*p*(1→	1	102.66	4.38	4.38 (H1)	3.32 (c: H3)
	2	72.85	3.36	3.36 (H2)	
	3			3.58 (H3)	
	4			ND	
	5			(-)	
	6			(-)	
b: →3)β-D-Gal*p*(1→	1	102.35	4.39	4.39 (H1)	3.39 (d: H2)
	2	73.23	3.19	3.19 (H2)	
	3	68.00	3.35	3.35 (H3)	
	4			ND	
	5			(-)	
	6			(-)	
c: →2,3)β-D-Glc*p*(1→	1	102.53	4.58	4.58 (H1)	3.35 (b: H3)
	2	73.38	3.21	3.21 (H2)	3.35 (b: H3)
	3	75.50	3.32	3.32 (H3)	3.35 (b: H3)
	4			ND	
	5			(-)	
	6			(-)	
d: β-D-Glc*p*(1→	1	101.37	4.60	4.60 (H1)	
	2	72.92	3.39	3.39 (H2) (+)	
	3			3.61 (H3) (+)	
	4			ND	
	5			(-)	
	6			(-)	
e: →2)β-D-Glc*p*(1→	1	102.77	4.64	4.64 (H1)	3.21 (c: H2) 3.35 (b: H3)
	2	69.50	3.24	3.24 (H2)	3.35 (b: H3)
	3			ND	
	4			ND	
	5		+	(-)	
	6			(-)	

ND: not determined; (-): cannot be assigned.

## Data Availability

The original contributions presented in the study are included in the article. Further inquiries can be directed to the corresponding author.
